# Future-Ready Care: Integrating Geroscience into Health Professions Training

**DOI:** 10.1093/geroni/igaf122.2338

**Published:** 2025-12-31

**Authors:** Gregory Hartley, Patricia Cuff, Cathy Maxwell

**Affiliations:** University of Miami, Coral Gables, Florida, United States; National Academies of Sciences, Engineering, and Medicine, Washington, District of Columbia, United States; University of Utah, Salt Lake City, Utah, United States

## Abstract

With increasing life expectancy and a growing aging population, healthcare systems must shift from a reactive, disease-centered approach to one that promotes healthy aging. It is the role of health professionals and their educators to move society in this direction. Such a transformation requires a unified approach to integrating principles of geroscience, interprofessional collaboration, and proactive aging strategies. A national call to action (unpublished National Academy of Medicine Perspective), collectively written by health professional educators from six health professions, directs colleagues on steps they can take to advance the principles and promote provider competence in the care of older adults. Calls to action were developed within three domains: Shifting Societal Norms; Focusing on Healthspan; and Educating a Competent Workforce. Each domain promotes four calls to action, with the top priorities listed here. • To shift societal norms, health professionals and educators must advocate for policy reforms that incentive a holistic approach to healthy aging. Demonstrating the financial return on investment will be key to successfully promoting policy reforms. • To influence individual patients’ lives, health professional providers should become educated on georscience and healthspan principles, and effectively communicate evidence to patients. Those who have limited understanding of the aging process should pursue professional development in geroscience and caring for older patients. • To educate the future generation of care providers, educators must incorporate into curricula, evidence-based data and information on geroscience and best practices in caring for older adults. Educating interprofessionally will strengthen learners’ understanding of the wider health system.

